# Association between the composite dietary antioxidant index and risk of endometriosis in women: a national population-based study

**DOI:** 10.3389/fnut.2025.1549948

**Published:** 2025-03-26

**Authors:** Yanmei Yu, Jianan Sun, Dandan Wang, Miaomiao Xing, Yanqi Yang

**Affiliations:** ^1^The Fourth School of Clinical Medicine, Harbin Medical University, Harbin, China; ^2^School of Mechatronics Engineering, Harbin Institute of Technology, Harbin, China; ^3^Jilin Hospital of Integrated Traditional Chinese and Western Medicine, Changchun, China; ^4^Department of Obstetrics and Gynecology, The Fourth Affiliated Hospital of Harbin Medical University, Harbin, China

**Keywords:** composite dietary antioxidant index, endometriosis, women, NHANES, crosssectional

## Abstract

**Background:**

Composite dietary antioxidant index (CDAI) has been found protective to women’s health. However, the association between CDAI level and the risk of endometriosis in women is unclear.

**Methods:**

A total of 4,153 women from the National Health and Nutrition Examination Survey (NHANES) 2001–2006 were included in this cross-sectional study. We evaluated the association between CDAI level and the risk of endometriosis using three logistic regression models and restricted cubic spline. Stratified and sensitivity analyses were also performed.

**Results:**

Logistic regression analysis found that CDAI level was inversely associated with the development of endometriosis. The associated odds ratio (OR) for each SD increase in CDAI was 0.98 [95%CI: 0.96–0.99]. After dividing the CDAI level into four quartiles, we found that compared with the CDAI level in Q1 (−1.89, −1.79), the ORs [95%CI] associated with endometriosis in Q2 (−1.79, −0.69), Q3 (−0.69, 1.42) and Q4 (1.42, 47.92) were 0.94 [0.87, 1.03], 0.95 [0.88, 1.04] and 0.83 [0.77, 0.91], respectively, with p trend<0.001. Restricted cubic spline showed a negative dose–response relationship between CDAI level and endometriosis risk. In addition, the protective effect of CDAI on endometriosis was more obvious in women aged 30–39 years (OR = 0.83, 95% CI = 0.69–0.99), gave relatively more births (OR = 0.82, 95% CI = 071–0.93), lower family income (OR = 0.69, 95% CI = 0.54–0.88), Non-Hispanic Black (OR = 0.72, 95% CI = 0.58–0.89), less educated (OR = 0.69, 95% CI = 0.52–0.91), smoker (OR = 0.74, 95% CI = 0.61–0.89), alcohol drinker (OR = 0.86, 95% CI = 0.77–0.97), overweight or obese (OR = 0.76, 95% CI = 0.60–0.97), and hypertensive (OR = 0.72, 95% CI = 0.60–0.87).

**Conclusion:**

Our findings may provide valuable insights into the primary prevention of endometriosis in women and further prospective studies are warranted.

## 1 Introduction

Endometriosis is defined as the presence of endometrial-like glands and stroma outside the uterus. It is estimated that this disease affects approximately 10% of women (1, 2) and is prone to serious complications, including dysmenorrhea and chronic pelvic pain (3). In addition, endometriosis can also lead to adverse prognoses, such as infertility, ovarian cancer, cardiovascular disease, and skin melanoma (4–6). The onset of endometriosis may be related to hormonal, neural and immune factors, lifestyle, diet, etc. In studies examining the association between diet and endometriosis, consumption of fruits and vegetables, fish oil, dairy products rich in calcium and vitamin D, and omega-3 fatty acids has been suggested to potentially reduce the risk of endometriosis (7–10). Conversely, intake of products high in trans-unsaturated fatty acids, general fat consumption, and intake of beef and other red meats, as well as alcohol, have been associated with an increased risk of endometriosis (7, 11, 12). However, no definitive correlations have been established between these foods and the risk of endometriosis. Further research is warranted to fully elucidate the impact of diet on the risk of developing endometriosis and to inform preventive strategies targeting this modifiable risk factor.

A large amount of evidence shows that vitamin and mineral supplements have a protective effect on women’s health (13–15). Antioxidants (such as vitamin A or carotenoids, vitamins C and E) can prevent oxidative stress in cells, thereby reducing cell damage and reducing the occurrence of diseases (16). A prospective cohort study of 34,492 postmenopausal women by Yochum et al. found that vitamin E in food had a protective effect on death caused by stroke (17). Osganian et al. conducted a prospective study and showed that women who took vitamin C supplements had a lower risk of coronary heart disease (18). However, a systematic review from the United States Preventive Services Task Force (USPSTF) found that supplementation of a single nutrient did not have a significant impact on preventing chronic diseases (cardiovascular disease, cancer, etc.) in adults (19). Therefore, a large number of studies suggest that people can supplement a variety of complex nutrients to maintain health (20, 21).

The composite dietary antioxidant index (CDAI) is a comprehensive score used to assess the total antioxidant capacity (TAC) of an individual’s diet. It is based on various dietary vitamins and minerals with antioxidant effects, including vitamins A, C, E and carotene, as well as the minerals selenium and zinc (22, 23). Previous studies have found that CDAI, as a new dietary health score indicator, is closely related to women’s health. A cross-sectional study by Shen et al. showed that there is a nonlinear negative correlation between CDAI and infertility in American women (24). Moreover, Li et al. performed a cross-sectional study on young American women and suggested that CDAI was negatively correlated with migraine attacks, and a higher CDAI may be a protective factor for preventing migraine attacks (24). Rumiris et al. conducted a randomized, double-blind, placebo-controlled daily antioxidant supplementation trial, and the results showed that compared with iron and folic acid supplementation alone, antioxidant supplementation in pregnant women with low antioxidant status was associated with better maternal and perinatal outcomes (25). Another cross-sectional study by Zhao et al. on Americans found that CDAI was negatively correlated with depression in overweight and obese adults, especially among women (26). However, the relationship between CDAI and endometriosis in women remains unclear.

Therefore, this study aimed to explore the relationship between CDAI and endometriosis in women using data from the National Health and Nutrition Examination Survey (NHANES), a large-scale and representative U.S. population survey. The results of this study may provide valuable insights into the role of CDAI levels in the occurrence of endometriosis in women, thereby providing important reference value for the primary prevention of endometriosis in female population worldwide.

## 2 Materials and methods

### 2.1 Data source

NHANES was initiated by the National Center for Health Statistics (NCHS) to construct a nationally representative health data of the American population. The survey uses a complex, multi-stage, stratified probability sampling, cross-sectional design to collect information on approximately 5,000 people each year. The survey began sampling in 1999 and releases data every 2 years. The research design of NHANES has been approved by the NCHS Research Ethics Review Board, and the study obtained informed consent from all participants. The NHANES survey data, detailed survey operation manual, informed consent form, and manuals for each sampling period are publicly available on the NHANES website.^1^

### 2.2 Study participants

**Figure 1:**
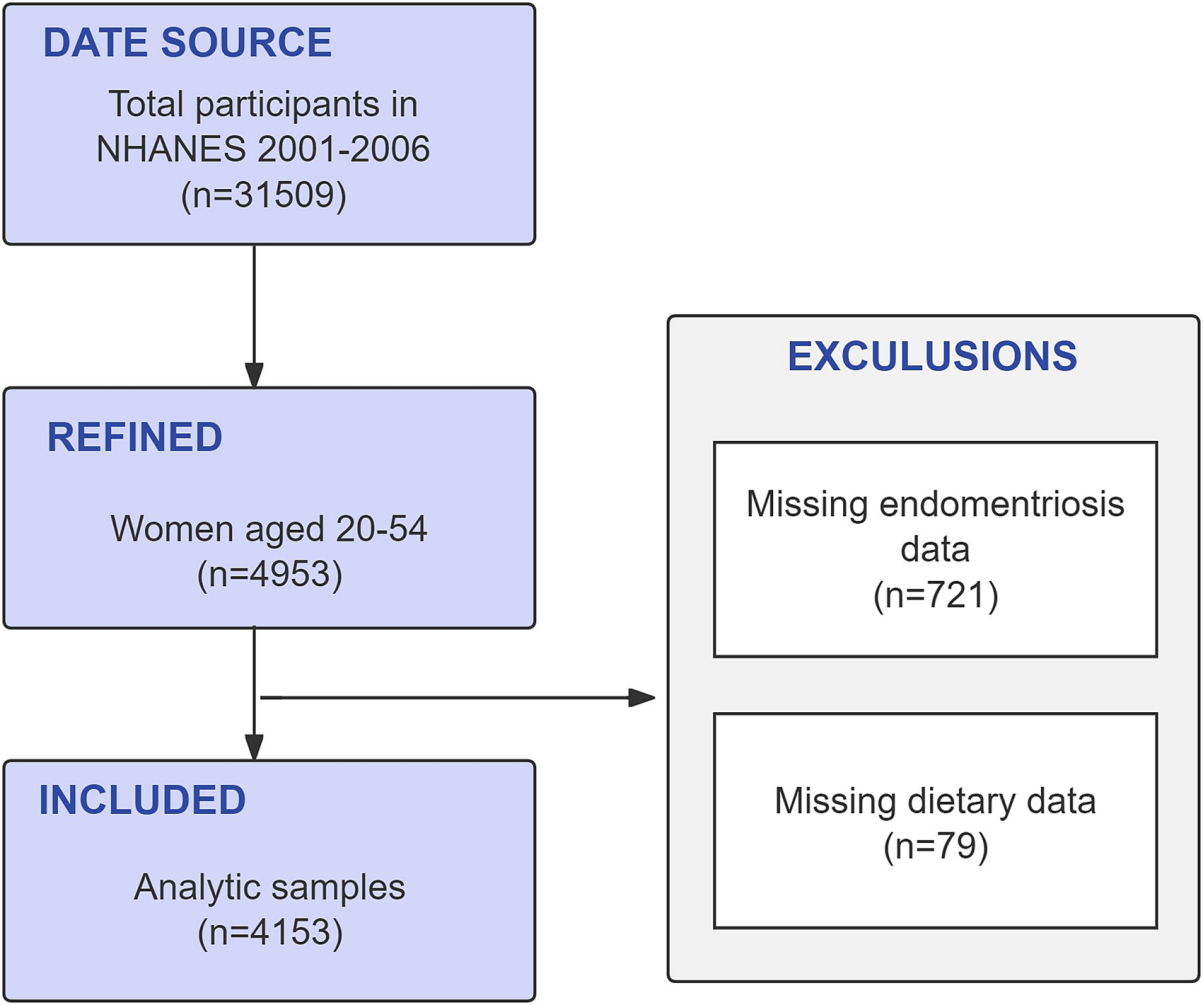
Flowchart of the study design. NHANES, National Health and Nutrition Examination Survey.

Since some years of the NHANES survey did not include assessments of endometriosis history or diet, we included data from subjects from 2001 to 2006, with a total of 31,509 people participating in the survey. The inclusion criteria for the subjects in this study were: (a) Women aged 20–54 years old; (b) With information about endometriosis diagnosis; (c) With 24-h dietary recall on the first and second day. After excluding samples that did not meet the above inclusion criteria, this study included 4,153 female subjects. The specific exclusion process is shown in Figure 1.

### 2.3 Outcome ascertainment

Our study outcome was the history of endometriosis diagnosis, which was assessed using the NHANES Reproductive Health Questionnaire (RHQ), which assesses women’s reproductive health issues, including menstrual history, pregnancy history, breastfeeding, use of oral contraceptives and hormone replacement therapy, and other related conditions. We used the RHQ question “Has a doctor or other health professional ever told that had endometriosis? (Endometriosis is a disease in which the tissue that forms the lining of the uterus/womb attaches to other places, such as the ovaries, fallopian tubes, or abdominal cavity)” to collect whether the subjects had endometriosis. Participants who answered “yes” to this question was considered to be diagnosed with endometriosis.

### 2.4 CDAI measurement

The dietary assessment data of the subjects was collected from the dietary section of NHANES, which assessed the subjects’ 24-h dietary recall data for two non-consecutive days and collected information on the participants’ food intake. The dietary recall interview on the first day of the subject was conducted at the Mobile Examination Center (MEC), and the dietary recall on the second day was conducted by telephone 3 to 10 days after the completion of the first recall interview. We calculated the average daily dietary intake based on the dietary recall data of these 2 days, and calculated the CDAI level of all subjects based on the study of Wright et al. (27). Specifically, the CDAI calculation is based on the sum of the average daily standardized intake of zinc, selenium, carotenoids, vitamin A, vitamin C, and vitamin E. The standardized intake is calculated by subtracting the mean value from the intake of the food and dividing it by the standard deviation. The specific calculation formula of CDAI is:


$$\mathrm{CDAI}=\overset{n=6}{\underset{t=1}{\sum }}\left(\mathrm{Individual}\;\mathrm{Intake}-\mathrm{Mean}\right)/SD\text{.}$$

### 2.5 Covariates

To reduce potential confounding bias in the analysis, we selected the following covariates based on previous relevant studies and clinical significance: age distribution (years) (20–29, 30–39, 40–49, 50–54), race (Mexican American, other Hispanic, Non-Hispanic White, Non-Hispanic Black, other race-including multi-racial), education level (<=HS graduate, HS graduate, some college or associate degree, college graduate or above), marital status (married, widowed, divorced, separated, never married, living with partner), ratio of family income to poverty (<1.3, 1.3–3.5, > = 3.5), BMI (kg/m2) (< 24.9, 25–29.9, 30–34.9, ≥35), drinking (yes or no), smoking—cigarette use (never, past, current smoker), number of live births (0, 1, 2, > = 3), hypertension (yes or no), diabetes (yes or no), coronary heart disease (yes or no), antihypertensive medication use (yes or no), antidiabetic medication use (yes or no), anticholesterol medication use (yes or no), and female hormone use (yes or no). Supplementary Table 1 provides a detailed description of these covariate assessments.

### 2.6 Statistical analysis

First, descriptive statistics were performed. Categorical variables were described as frequency and percentage, and the chi-square test or Fisher’s exact test was used to compare the endometriosis patient group with the non-patient group. Numerical variables that conformed to the normal distribution were described as mean (± standard deviation [SD]), and the independent sample t test was used for inter-group comparison. Numerical variables that were not normally distributed were described as median [first quartile (P25) and third quartile (P75)], and the Wilcoxon test was used for group comparison. Second, three logistic regression models were used to explore the relationship between CDAI and endometriosis. Model A only adjusted for age; Model B adjusted for age, race, education level, marital status, the ratio of family income to poverty, number of live births and BMI; Model C further adjusted for alcohol use, smoking—cigarette use, hypertension, diabetes, congestive heart failure, and coronary heart disease on the basis of Model B. In addition, we also used the restricted cubic spline (RCS) method to explore the dose–response relationship between CDAI level and the risk of endometriosis. We further conducted subgroup analysis for age distribution (years), number of live births, ratio of family income to poverty, race, education level, marital status, smoking cigarette use, alcohol use, BMI (kg/m2), coronary heart disease, hypertension, diabetes. We used product interaction terms to measure whether there was an interaction effect between CDAI and each covariate. Finally, we also conducted several sensitivity analyses to evaluate the robustness of the research results. Specifically, we first excluded participants who had previously used female hormones, and verified the relationship between CDAI and endometriosis based on the above three logistic regression models. In addition, we excluded participants who used antihypertensive drugs, hypoglycemic drugs, and lipid-lowering drugs, respectively, and also took the above steps to verify the findings of this study. Finally, we excluded individuals with any missing values for the variables involved in this study. All *p* values reported in this study were two-sided tests, and the significance level was set at *p* < 0.05. We used recommended NHANES weight parameters to calculate weighted characteristics. All statistical analyses were performed using R software (version 4.4.1).

## 3 Results

### 3.1 Population characteristics

A total of 4,153 adult women were included in this study, with a mean age of 35.6 ± 10.0 years, 21.9% Mexican American, 4.1% other Hispanic, 48.0% Non-Hispanic White, and 21.3% Non-Hispanic Black. Among them, 288 were diagnosed with endometriosis. The median CDAI level (P25, P75) of the endometriosis patient group was −1.28 [−1.81, 0.42], which was lower (*p* = 0.006) than that of non-endometriosis patients. Compared with the non-endometriosis group, the intake of vitamin E (*p* = 0.047), vitamin A (*p* = 0.010), beta-carotene (*p* = 0.003), vitamin C (*p* = 0.004), and selenium (*p* = 0.014) was significantly lower in the endometriosis group. In addition, the two groups have different differences in age (*p* < 0.001), education level (*p* = 0.002), and family income (*p* < 0.001). There were significant differences in, marital status (*p* < 0.001), drinking (*p* = 0.018), smoking history (*p* = 0.001), suffering from hypertension (*p* < 0.001), and taking estrogen drugs (*p* < 0.001; Table 1).

**Table 1:** Characteristics of study participants from NHANES 2001–2006^a^.

Variables	Overall	Endometriosis	Non-endometriosis	*p*-value
	(*n* = 4,153)	(*n* = 288)	(*n* = 3,865)	
Weighted sample size	63,131,684	5,600,037	57,531,647	
Age, mean ± SD	35.6 ± 10.0	40.5 ± 8.5	35.3 ± 10.1	<0.001
Age distribution (years), *n* (%)				<0.001
20–29	1,416 (34.1)	37 (12.8)	1,379 (35.7)	
30–39	1,154 (27.8)	84 (29.2)	1,070 (27.7)	
40–49	1,092 (26.3)	117 (40.6)	975 (25.2)	
50–54	491 (11.8)	50 (17.4)	441 (11.4)	
Race, *n* (%)				<0.001
Mexican American	909 (21.9)	24 (8.3)	885 (22.9)	
Other Hispanic	171 (4.1)	6 (2.1)	165 (4.3)	
Non-Hispanic White	1992 (48.0)	195 (67.7)	1797 (46.5)	
Non-Hispanic Black	885 (21.3)	55 (19.1)	830 (21.5)	
Other Race-Including Multi-Racial	196 (4.7)	8 (2.8)	188 (4.9)	
Education level, *n* (%)				0.002
<=HS graduate	963 (24.1)	41 (14.7)	922 (24.8)	
HS graduate	945 (23.6)	78 (28.1)	867 (23.3)	
Some college or associate degree	1,191 (29.8)	90 (32.4)	1,101 (29.6)	
College graduate or above	900 (22.5)	69 (24.8)	831 (22.3)	
Marital status, *n* (%)				<0.001
Married	2,318 (55.8)	180 (62.5)	2,138 (55.3)	
Widowed	59 (1.4)	6 (2.1)	53 (1.4)	
Divorced	365 (8.8)	38 (13.2)	327 (8.5)	
Separated	164 (3.9)	14 (4.9)	150 (3.9)	
Never married	858 (20.7)	34 (11.8)	824 (21.3)	
Living with partner	388 (9.3)	16 (5.6)	372 (9.6)	
Ratio of family income to poverty, *n* (%)				<0.001
<1.3	1,120 (28.2)	56 (20.0)	1,064 (28.9)	
1.3to3.5	1,464 (36.9)	91 (32.5)	1,373 (37.2)	
> = 3.5	1,382 (34.8)	133 (47.5)	1,249 (33.9)	
BMI (kg/m2), mean ± SD	28.86 ± 7.38	28.60 ± 6.90	28.88 ± 7.42	0.684
BMI (kg/m2), *n* (%)				0.727
< 24.9	1,417 (34.5)	106 (36.9)	1,311 (34.3)	
25–29.9	1,175 (28.6)	77 (26.8)	1,098 (28.7)	
30–34.9	786 (19.1)	57 (19.9)	729 (19.1)	
≥35	731 (17.8)	47 (16.4)	684 (17.9)	
Alcohol use, *n* (%)	2,533 (61.0)	195 (67.7)	2,338 (60.5)	0.018
Smoking—cigarette use, *n* (%)				0.001
Never	2,562 (61.7)	150 (52.1)	2,412 (62.4)	
Past	672 (16.2)	53 (18.4)	619 (16.0)	
Current	917 (22.1)	85 (29.5)	832 (21.5)	
Hypertension, *n* (%)^b^	744 (18.0)	85 (29.5)	659 (17.1)	<0.001
Diabetes, *n* (%)^c^	195 (4.7)	11 (3.8)	184 (4.8)	0.553
Coronary Heart Disease, *n* (%)	28 (0.7)	4 (1.4)	24 (0.6)	0.245
Antihypertensive medication use, *n* (%)^d^	504 (67.7)	59 (69.4)	445 (47.5)	0.820
Antidiabetic medication use, *n* (%) ^d^	125 (46.8)	9 (45.0)	116 (47.0)	1.000
Anticholesterol medication use, *n* (%)^d^	224 (33.8)	27 (32.9)	197 (34.0)	0.951
Female hormone use, *n* (%)^e^	485 (11.7)	110 (38.3)	375 (9.7)	<0.001
Vitamin E, median[IQR]^f^	−0.28 [−0.37,0.01]	−0.32 [−0.37,-0.11]	−0.28 [−0.37,0.02]	0.047
Vitamin A, median[IQR]^f^	−0.25 [−0.25,0.19]	−0.25 [−0.25,-0.04]	−0.25 [−0.25,0.20]	0.010
Beta-carotene, median[IQR]^f^	−0.14 [−0.14,-0.12]	−0.14 [−0.14,-0.13]	−0.14 [−0.14,-0.12]	0.003
Vitamin C, median[IQR]^f^	−0.27[−0.27,-0.22]	−0.27[−0.27,-0.25]	−0.27[−0.27,-0.22]	0.004
Zine, median[IQR]^f^	−0.23 [−0.33,0.17]	−0.27 [−0.33,0.01]	−0.22 [−0.33,0.18]	0.109
Selenium, median[IQR]^f^	−0.33 [−0.46,0.33]	−0.39 [−0.46,0.18]	−0.31 [−0.46,0.34]	0.014
CDAI, median[IQR]	−0.69 [−1.79,1.42]	−1.28 [−1.81,0.42]	−0.65 [−1.79,1.49]	0.006

BMI, body mass index; CDAI, composite dietary antioxidant index; NHANES, National Health and Nutrition Examination Survey; ^a^All estimates accounted for sample weights and complex survey designs, and percentages and means were adjusted for survey weights of NHANES. ^b^Hypertension was defined based on self-reported information. ^c^Diabetes was defined as self-reported diabetes. ^d^Antihypertensive, Anticholesterol, or antidiabetic medication use were defined as individuals currently taking them. ^e^Female hormone use was defined as individuals who had ever used any forms of female hormones, including pills, creams, patches, and injectables, but not for birth control or infertility purposes. ^f^Vitamin E, Vitamin A, Beta-carotene, Vitamin C, Zine, Selenium, were reported as standardized scores.

### 3.2 Association between CDAI level and risk of endometriosis

Table 2 illustrates the association between CDAI level and risk of endometriosis in women using a multivariate logistic regression model. In model A, we observed that continuous CDAI level was negatively associated with the occurrence of endometriosis, and the associated odds ratio (OR) for each SD increase in CDAI was 0.98 [95% CI: 0.96–0.99]. After dividing CDAI levels into quartiles, it was found that compared with CDAI levels in Q1 (−1.89, −1.79), Q2 (−1.79, −0.69), Q3 (−0.69, 1.42) and Q4 (1.42, 47.92) were associated with endometriosis with ORs of 0.94 [0.87, 1.03], 0.95 [0.88, 1.04] and 0.83 [0.77, 0.91], respectively, with a *p* trend<0.001.

**Table 2:** Associations between CDAI Levels and the Risks of Endometriosis in Women aged 20–54 years ^a^.

CDAI	Model A		Model B		Model C	
	OR [95% CI]	*p* value	OR [95% CI]	*p* value	OR [95% CI]	*p* value
As continuous (per SD)	0.98 [0.96,0.99]	0.001	0.98 [0.96,0.99]	<0.001	0.98 [0.96,0.99]	<0.001
Interquartile						
Quartile 1 [−1.89,-1.79]	Reference		Reference		Reference	
Quartile 2 [−1.79,-0.69]	0.94 [0.87,1.03]	0.210	0.93 [0.85,1.02]	0.106	0.95 [0.86,1.04]	0.266
Quartile 3 [−0.69,1.42]	0.95 [0.88,1.04]	0.303	0.94 [0.86,1.02]	0.160	0.95 [0.86,1.04]	0.305
Quartile 4 [1.42,47.92]	0.83 [0.77,0.91]	<0.001	0.84 [0.77,0.92]	<0.001	0.86 [0.78,0.94]	0.001
p-trend	<0.001		<0.001		0.005	

CDAI, composite dietary antioxidant index; CI, confidence interval; OR, odds ratio. ^a^The associations between CDAI levels and the risks of and the Risks of Endometriosis are presented as ORs (95% CI). Model A adjusted for age. Model B adjusted for age, race, education level, marital status, the ratio of family income to poverty, number of live births and BMI. Model C further adjusted for alcohol use, smoking—cigarette use, hypertension, diabetes, congestive heart failure, coronary heart disease based on Model B.

In model B, higher CDAI levels were associated with a lower risk of endometriosis (OR = 0.98, 95% CI: 0.96–0.99) after adjusting for age, race, education level, marital status, the ratio of family income to poverty, number of live births and BMI. Similarly, compared with Q1 level, the OR of CDAI level in Q2, Q3 and Q4 were 0.93 [0.85, 1.02], 0.94 [0.86, 1.02] and 0.84 [0.77, 0.92], respectively, with p trend<0.001.

**Figure 2:**
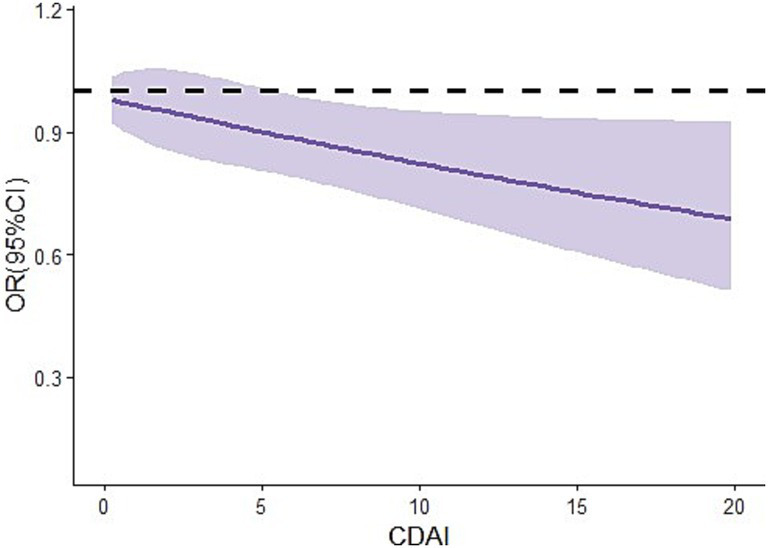
Dose–response relationship between CDAI levels and the risks of endometriosis in women aged 20–54 years. CI, confidence interval; OR, odds ratio; CDAI, composite dietary anti-oxidant index.

In addition, after additional adjustment for alcohol use, smoking-cigarette use, hypertension, diabetes, congestive heart failure, coronary heart disease based on model B, we obtained similar findings in model C, with the OR of Q4 being 0.86 [95% CI: 0.78, 0.94] compared with Q1. In addition, the results of the RCS curve showed that there was a negative dose–response relationship between CDAI level and the risk of endometriosis (Figure 2).

### 3.3 Subgroup analysis

The results of subgroup analysis based on different categorical variables showed that compared with low CDAI, high CDAI was significantly associated with a lower risk of endometriosis in the following subgroups: 30–39 years (OR = 0.83, 95% CI: 0.69–0.99), number of live births = 2 (OR = 0.79, 95% CI: 0.64–0.98) or ≥ 3 (OR = 0.82, 95% CI: 0.71–0.93), ratio of family income to poverty<1.3 (OR = 0.69, 95% CI: 0.54–0.88), Non-Hispanic Black (OR = 0.72, 95% CI: 0.58–0.89), education level ≤ HS graduate (OR = 0.69, 95% CI: 0.52–0.91), current smoker (OR = 0.74, 95% CI: 0.76–0.93), past alcohol drinker (OR = 0.86, 95% CI: 0.77–0.97), BMI 25–29.9 kg/m2 (OR = 0.80, 95% CI: 0.67–0.97) or ≥ 35 kg/m2 (OR = 0.76, 95% CI: 0.60–0.97), no coronary heart disease (OR = 0.86, 95% CI: 0.78–0.95), with hypertension (OR = 0.72, 95% CI: 0.60–0.87), no diabetes (OR = 0.86, 95% CI: 0.78–0.94; Table 3).

**Table 3:** Subgroup analysis of associations between CDAI levels and the risks of endometriosis^a^.

	ORs (95% CI)					
	As continuous (per SD)	Quartile 1	Quartile 2	Quartile 3	Quartile 4	*p* for interaction
Age distribution (years)						
20–29	0.99 [0.95,1.02]	Ref.	0.98 [0.76,1.26]	1.08 [0.84,1.38]	0.81 [0.62,1.05]	0.056
30–39	0.98 [0.95,1.00]		0.92 [0.77,1.10]	0.96 [0.81,1.14]	0.83 [0.69,0.99]^*^	
40–49	0.98 [0.96,1.00]		0.99 [0.86,1.15]	0.93 [0.80,1.08]	0.95 [0.82,1.11]	
50–54	0.94 [0.90,0.98]^**^		0.98 [0.78,1.23]	0.99 [0.79,1.25]	0.80 [0.62,1.02]	
Number of live births						
0	0.97 [0.94,1.00]	Ref.	0.84 [0.66,1.08]	0.92 [0.72,1.18]	0.87 [0.68,1.11]	0.041
1	1.02 [0.98,1.04]		1.07 [0.84,1.37]	1.02 [0.79,1.30]	1.04 [0.81,1.33]	
2	0.97 [0.94,0.99]^*^		0.95 [0.77,1.17]	1.02 [0.83,1.26]	0.79 [0.64,0.98]^*^	
> = 3	0.97 [0.95,0.99]^**^		0.93 [0.82,1.07]	0.90 [0.79,1.04]	0.82 [0.71,0.93]^**^	
Ratio of family income to poverty						
<1.3	0.97 [0.94,1.00]	Ref.	0.83 [0.66,1.04]	0.93 [0.73,1.17]	0.69 [0.54,0.88]^*^	0.055
1.3to3.5	0.96 [0.93,0.98]^**^		1.06 [0.89,1.25]	1.01 [0.85,1.19]	0.97 [0.73,1.03]	
> = 3.5	0.99 [0.97,1.01]		0.91 [0.80,1.03]	0.91 [0.80,1.04]	0.91 [0.80,1.04]	
Race						
Mexican American	0.95 [0.91,0.99]^*^	Ref.	1.22 [0.90,1.65]	1.25 [0.91,1.70]	0.97 [0.70,1.33]	0.006
Non-Hispanic Black	0.96 [0.93,0.99]^*^		0.68 [0.54,0.85]^***^	0.71 [0.57,0.88]^**^	0.72 [0.58,0.89]^**^	
Non-Hispanic White	0.98 [0.97,0.99]^*^		1.01 [0.90,1.13]	1.03 [0.91,1.15]	0.90 [0.80,1.02]	
Other Hispanic	1.04 [0.94,1.14]		1.01 [0.47,2.15]	0.94 [0.44,1.97]	0.99 [0.49,2.04]	
Other Race-Including Multi-Racial	0.96 [0.83,1.14]		0.38 [0.01,4.89]	0.27 [0.01,3.62]	0.20 [0.00,1.58]	
Education level						
<=HS graduate	0.95 [0.91,0.98]^*^	Ref.	0.80 [0.61,1.04]	0.87 [0.67,1.14]	0.69 [0.52,0.91]^*^	0.014
College graduate or above	1.00 [0.98,1.02]		0.95 [0.80,1.14]	1.04 [0.87,1.23]	0.96 [0.80,1.14]	
HS graduate	0.97 [0.95,1.00]		1.03 [0.85,1.24]	0.92 [0.76,1.12]	0.85 [0.69,1.04]	
Some college or associate degree	0.96 [0.94,0.99]^**^		0.98 [0.83,1.15]	0.97 [0.82,1.14]	0.87 [0.74,1.03]	
Marital status						
Married/Living with partner	0.98 [0.97,1.00]	Ref.	0.94 [0.85,1.06]	0.94 [0.84,1.06]	0.88 [0.78,0.99]*	0.064
Widowed/Divorced/Separated/Never married	0.96 [0.94,0.98]**		0.93 [0.79,1.10]	0.95 [0.81,1.12]	0.77 [0.65,0.91]**	
Smoking—cigarette use						
current smoker	0.97 [0.94,0.99]^*^	Ref.	0.88 [0.74,1.06]	0.95 [0.79,1.15]	0.74 [0.61,0.89]^**^	0.256
never smoker	0.98 [0.96,0.99]^*^		0.99 [0.88,1.12]	0.97 [0.86,1.10]	0.90 [0.80,1.03]	
past smoker	0.98 [0.95,1.01]		0.86 [0.69,1.09]	0.93 [0.73,1.17]	0.91 [0.72,1.16]	
Alcohol use						
never drinker	0.98 [0.96,1.00]	Ref.	0.86 [0.73,1.02]	0.98 [0.84,1.15]	0.86 [0.73,1.01]	0.739
past drinker	0.98 [0.96,0.99]^*^		0.99 [0.89,1.12]	0.94 [0.84,1.06]	0.86 [0.77,0.97]^*^	
BMI (kg/m2)						
< 24.9	0.98 [0.97,1.00]	Ref.	1.03 [0.89,1.19]	1.04 [0.89,1.21]	0.95 [0.81,1.11]	0.251
25–29.9	0.97 [0.95,0.99]^*^		0.78 [0.64,0.92]^**^	0.82 [0.68,0.99]^*^	0.80 [0.67,0.97]^*^	
30–34.9	0.98 [0.95,1.01]		1.13 [0.91,1.40]	1.17 [0.94,1.44]	0.86 [0.69,1.08]	
≥35	0.96 [0.93,0.99]^*^		0.94 [0.74,1.20]	0.86 [0.67,1.10]	0.76 [0.60,0.97]^*^	
Coronary Heart Disease						
Yes	0.84 [0.67,1.01]	Ref.	0.51 [0.23,1.10]	0.20 [0.08,0.47]^***^	0.47 [0.19,1.09]	0.089
No	0.98 [0.97,0.99]^*^		0.96 [0.87,1.05]	0.97 [0.88,1.07]	0.86 [0.78,0.95]^**^	
Hypertension						
Yes	0.97 [0.95,0.99]^**^	Ref.	0.75 [0.63,0.90]^**^	0.82 [0.68,0.99]^*^	0.72 [0.60,0.87]^***^	0.486
No	0.98 [0.96,0.99]^*^		1.04 [0.93,1.16]	1.02 [0.91,1.14]	0.91 [0.82,1.02]	
Diabetes						
Yes	0.97 [0.00,0.00]	Ref.	0.91 [0.00,0.00]	0.72 [0.00,0.00]	1.01 [0.00,0.00]	0.315
No	0.97 [0.00,0.00]		0.95 [0.86,1.04]	0.96 [0.87,1.05]	0.86 [0.78,0.94]^*^	

CDAI, composite dietary antioxidant index; BMI, body mass index; CI, confidence interval; OR, odds ratio. ^a^The associations between CDAI levels and the risks of and the Risks of Endometriosis are presented as ORs (95% CI). Model adjusted for age, race, education level, marital status, the ratio of family income to poverty, number of live births BMI, alcohol use, smoking—cigarette use, hypertension, diabetes, congestive heart failure, coronary heart disease. ^*^p < 0.05; ^**^p < 0.01; ^***^p < 0.001.

### 3.4 Sensitivity analysis

After excluding participants who had ever used female hormones, antihypertensive drugs, lipid-lowering drugs, and antidiabetic drugs respectively, high levels of CDAI still had a significant protective effect on women with endometriosis (Supplementary Tables 2–5), indicating that the effect of CDAIs on the risk of endometriosis is not interfered with by the use of these drugs. In addition, after excluding individuals with missing covariates, the association between CDAI and the risk of endometriosis still holds (Supplementary Table 6), which is consistent with the main research results and illustrates the robustness of the results of this study.

## 4 Discussion

Our study is the first to investigate the relationship between CDAI and the risk of endometriosis in a nationally representative adult female population in the United States. First, we observed that the risk of endometriosis was lower in women with higher CDAI levels, and this was still robust after controlling for all covariates. In addition, there was a negative dose–response relationship between the level of CDAI and the risk of endometriosis. We further found that the protective effect of high CDAI levels on endometriosis were more pronounced in females aged 30–39 years, gave relatively more births, lower family income, Non-Hispanic Black, less educated, current smoker, past alcohol drinker, overweight or obese, no coronary heart disease, with hypertension, no diabetes.

We found that CDAI is an important protective factor for the risk of endometriosis, indicating that an antioxidant diet can play an important role in the primary prevention of endometriosis. Although there is limited evidence that CDAI is associated with the risk of endometriosis. Some studies have found that dietary factors are associated with the risk of endometriosis. A case–control study conducted by Parazzini et al. found that consuming more green vegetables and fresh fruits can reduce the risk of endometriosis in women (7), which was verified in another literature review of 11 studies (28). A diet rich in green vegetables and fruits is rich in vitamin C, carotenoids, folic acid, and lycopene, and these micronutrients may help inhibit cell proliferation (29). It is worth noting that an antioxidant diet also has a protective effect on women who already have endometriosis. An observational study by Krabbenborg et al. on 157 patients with endometriosis showed that an antioxidant diet can improve the symptoms of endometriosis and improve the quality of life of patients (30). Supplementing relevant antioxidant diets can inhibit endometriosis-related symptoms in patients with endometriosis, which may be related to the anti-inflammatory, antioxidant, antiproliferative and immunomodulatory effects of antioxidant diets (31). In addition, Mier-Cabrera et al. found that after patients with endometriosis adopted a high-antioxidant diet, their antioxidant markers in peripheral blood were improved (32). Additionally, an antioxidant-rich diet confers protective effects against complications associated with endometriosis. For instance, antioxidants have been demonstrated to modulate oxidative stress and cellular signaling pathways in ovarian clear cell carcinoma, which may be of significant importance for the prevention and treatment of ovarian cancer (33). Antioxidants can also inhibit the development and progression of cervical cancer (34). Moreover, antioxidants provide protection against infertility in women, which is a common complication of endometriosis (35).

Important components of CDAI include vitamins A, C, E, carotene and important trace elements (zinc, selenium). These dietary elements play an important role in the occurrence and development of endometriosis. Pierzchalski et al. found that all-trans retinoic acid (ATRA) can promote hormonal changes (36) and inhibit endometriosis. In addition, some studies have found that ATRA can also reduce the level of IL-6, which is involved in the pathogenesis of endometriosis (37), including the migration and invasion of endometriotic cells (38). Studies have shown that vitamin A has the effect of preventing dioxin-induced tissue damage (39, 40). A population-based study by Zhou et al. found that women with endometriosis had lower vitamin A intake than women without endometriosis (41). Further, *β*-carotene is an important source of vitamin A (42). A large number of studies have found that β-carotene can prevent lipid peroxidation in cells and reduce free radical damage to DNA (43, 44). β-Carotene is also thought to enhance NK cell function (45). It is reported that approximately 50% of the vitamin A in the diet of the American population comes from β-carotene (46). These studies all suggest that vitamin A and carotene may protect against the development of endometriosis.

Vitamin C and E are two common antioxidants. Vitamin C, as a cofactor for a variety of essential enzymes, is involved in the synthesis of catecholamines and vasopressin (47) and in the hydroxylation of collagen (48, 49). Studies have demonstrated that vitamin C can inhibit the adhesion and growth of endometrial cells, thereby alleviating the symptoms of endometriosis (50). Vitamin E has anti-angiogenic and anti-inflammatory effects (51, 52). Vitamin E has been shown to reduce levels of free radicals and reactive oxygen species (ROS), thereby attenuating oxidative stress and inflammatory responses, which in turn alleviates pain associated with endometriosis. Additionally, vitamin E may modulate immune responses and decrease levels of inflammatory markers (53). Since both vitamins are involved in antioxidant processes, which are also the basis of the pathogenesis of endometriosis, these two vitamins may have some protective value against the development of endometriosis. Although vitamin C is primarily considered a micronutrient with a protective effect against endometriosis (54–56), some studies have shown that vitamin E is not associated with the risk of this disease (55). It is worth noting that some studies have found that the two vitamins have a synergistic effect, and their combined use may have a better antioxidant effect and be more beneficial for disease prevention (57).

Trace elements play an important role in the occurrence and development of endometriosis, especially zinc and selenium in CDAI. Zinc is involved in the composition of proteins in various construction, enzymatic and catalytic processes in cells, and helps maintain the body’s homeostasis; it also exists in the form of ions and acts as a signaling molecule (58). Studies have found that the concentration of MMP is positively correlated with the severity of endometriosis (59), and zinc deficiency may affect MMP, and estrogen levels may also affect MMP. In addition, zinc constitutes an important component of ZEB1 and ZEB2 molecules, which are involved in epithelial-mesenchymal transition (EMT) in the process of endometriosis and are associated with the severity of endometriosis. However, no studies have found that zinc deficiency may impair the expression of ZEB1 and ZEB2. In the studies of Messala et al. and Lai et al., the blood zinc levels of patients with endometriosis were measured to be 22% lower than those of healthy control women (60) and 43% lower (61), respectively, which is consistent with the findings of our study.

On the other hand, selenium is an important component of selenoproteins in the human body, which can function both as enzymes and as non-enzyme proteins (62). In endometriosis, selenium is positively correlated with glutathione peroxidase (63), which is an important component for regulating cellular oxidative stress. Therefore, there is a certain relationship between selenium and the occurrence of endometriosis. Singh et al. observed that lower selenium concentrations in follicular fluid were associated with an increased risk of endometriosis-related infertility compared with tubal infertility. In a study of patients with endometriosis, Guerrero et al. found that when patients took vitamin E, C, selenium and zinc at the same time, the severity of the disease decreased. In contrast, the lower the oral antioxidant nutrient intake of patients, the more severe the disease (64). Zinc and selenium are essential trace elements with significant immunomodulatory effects. Sahdeo et al. demonstrated that zinc, by forming complexes with various bioactive molecules, exhibits anti-inflammatory and antioxidant properties; zinc can exert anti-inflammatory effects by inhibiting the TLR4/NF-κB signaling pathway (65). Selenium, as an antioxidant, can modulate immune responses and reduce oxidative stress, thereby alleviating inflammation (65). It should be noted that other non-nutritive antioxidants can also play significant roles in the development of endometriosis. Curcumin can reduce the production of pro-inflammatory cytokines (such as TNF-*α*, IL-1, and IL-6) by inhibiting the NF-κB, JAK/STAT, and MAPK signaling pathways (66). Moreover, curcumin can activate endogenous antioxidant enzymes (such as superoxide dismutase [SOD] and glutathione peroxidase [GPx]), further enhancing its anti-inflammatory effects (65). Pázmándi et al. showed that gingerol can reduce inflammatory responses by inhibiting the NF-κB and PI3K/Akt/mTOR signaling pathways (67).

Finally, the association between CDAI and the risk of endometriosis is heterogeneous in different subgroups, which may be related to the different susceptibility of different groups to endometriosis. Most patients with endometriosis develop disease before the age of 45 (68). We found that the protective effect of CDAI on endometriosis is most significant among people aged 30–39, indicating that relatively younger individuals are more likely to benefit from the protection of an antioxidant diet. In addition, previous studies have found that black women have a lower incidence of endometriosis than white women (69). It was found that non-pregnant women have a higher risk of endometriosis, so the protective effect of antioxidant diet on these women might be smaller than that of women who have been pregnant (50, 70). Women who have been pregnant exhibit a lower risk of endometriosis due to the hormonal environment and immune regulation during pregnancy (71). In addition, women with higher education levels were reported to have a higher risk of endometriosis (72). However, we found a protective effect of an antioxidant diet against the development of endometriosis only in relatively low-educated women. No study has found a clear link between marital status and the risk of endometriosis (73). Our study found that CDAI has a protective effect on endometriosis in women with different marital statuses, indicating that this effect has nothing to do with whether they are single or not. The results of a meta-analysis showed that the risk of endometriosis between smoking and endometriosis was not significant (74), but our study only found a protective effect of CDAI on endometriosis among current smokers, indicating that there may be a certain interaction between these factors, which is worthy of further exploration. Another study found that there is a certain positive correlation between drinking alcohol and the occurrence of endometriosis (75). We found that the protective effect of CDAI on endometriosis was only among women with a history of drinking. Tang et al. found that compared with women of normal weight, obese women have a higher incidence of endometriosis (76). Our study found that the protective effect of CDAI is more obvious among women with high BMI or obesity. Overall, women diagnosed with endometriosis are more likely to develop coronary heart disease (77), hypertension, and hypercholesterolemia (78). A diagnosis of endometriosis is associated with an increased risk of developing type 2 diabetes in non-obese women and in women without a history of infertility or gestational diabetes (79). On the other hand, women with hypertension and hypercholesterolemia are also more likely to develop endometriosis (78), indicating that there is a certain interaction between endometriosis and the above diseases. This study found that the protective effect of CDAI on endometriosis is more obvious in women with hypertension, but without diabetes and coronary heart disease, the specific mechanism remains to be explored. However, this study has certain limitations. First, we used a cross-sectional design, so we cannot make clear causal inferences. Second, this study only included vitamin A, vitamin C, vitamin E, carotenoids, zinc and selenium in dietary components to calculate the level of CDAI, but did not consider additional nutrient supplements. The calculation of the Composite Dietary Antioxidant Index (CDAI) is based on two 24-h dietary recalls, which may not reflect the long-term dietary habits of the participants. Future studies could further evaluate the relationship between long-term dietary habits and endometriosis. In addition, only American female population was used in this study, so our findings cannot be extrapolated to all women in the world. Therefore, more research is needed, especially longitudinal and ethnically diverse studies to verify our findings.

## 5 Conclusion

Our study firstly explores the relationship between CDAI and the risk of endometriosis based on a large nationally representative female sample of childbearing age. We found that women with higher CDAI levels tend to have a lower incidence of endometriosis, and there is a negative dose–response relationship between CDAI levels and the risk of endometriosis. In addition, we also explored the differences in the protective effect of CDAI on the occurrence of endometriosis in different subgroups. A protective effect of CDAI on the risk of endometriosis was observed in our study, but it should be noted that, based on the cross-sectional design, causality cannot be fully established; it remains unclear whether an antioxidant-rich diet reduces the risk of endometriosis or if women with endometriosis simply have poorer diets. Prospective or interventional studies are required to confirm the causal nature of this relationship. In total, our results provide an important reference for the primary prevention of endometriosis.

## Data Availability

The datasets presented in this study can be found in online repositories. The names of the repository/repositories and accession number (s) can be found at: https://www.cdc.gov/nchs/nhanes/.

## Footnotes

^1^ https://www.cdc.gov/nchs/nhanes
