# Supplementary material for: Association between the composite dietary antioxidant index and risk of endometriosis in women: a national population-based study
Source: Front Nutr. 2025 Mar 26;12:1549948. doi: 10.3389/fnut.2025.1549948 (PMC11978645; doi:10.3389/fnut.2025.1549948)
Supplement: Supplementary file 1 [file Table_1.docx]

Supplementary Material

| Supplementary Table 1. Assessment of Covariates. | | | |
| --- | --- | --- | --- |
| Covariate | Assessment | Type | Range or Labels |
| Age | Best age in years of the sample person at time of screening. Individuals 85 and over are topcoded at 85 years of age. | Numerical | 20-54 |
| Race | Recode of reported race and ethnicity information. |  | Mexican American/Other Hispanic/Non-Hispanic White/Non-Hispanic Black/Other Race-Including Multi-Racial |
| Education level | What is the highest grade or level of school you completed or the highest degree you received? | Categorical | <=HS graduate/HS graduate/Some college or associate degree/College graduate or above |
| Marital status | Marital Status | Categorical | Married/Widowed/Divorced/Separated/Never married/Living with partner |
| Ratio of family income to poverty | Poverty income ratio (PIR) - a ratio of family income to poverty threshold | Numerical | 0-5 |
| BMI | Body Mass Index (kg/m^2^) | Numerical | 14.65-72.07 |
| Alcohol use | In any one year, you had at least 12 drinks of any type of alcoholic beverage? By a drink, I mean a 12 oz. beer, a 4 oz. glass of wine, or an ounce of liquor. | Categorical | Yes/No |
| Smoking—cigarette use | Have you smoked at least 100 cigarettes in your entire life? | Categorical | Yes/No |
| Hypertension | Have you ever been told by a doctor or other health professional that you had hypertension, also called high blood pressure? | Categorical | Yes/No |
| Diabetes | Other than during pregnancy, have you ever been told by a doctor or health professional that you have diabetes or sugar diabetes? | Categorical | Yes/No |
| Coronary Heart Disease | Has a doctor or other health professional ever told you that you had coronary heart disease? | Categorical | Yes/No |
| Antihypertensive medication use | Because of your hypertension, have you ever been told to take prescribed medicine? | Categorical | Yes/No |
| Antidiabetic medication use | Are you now taking diabetic pills to lower your blood sugar? These are sometimes called oral agents or oral hypoglycemic agents. | Categorical | Yes/No |
| Anticholesterol medication use | To lower your blood cholesterol, have you ever been told by a doctor or other health professional to take prescribed medicine? | Categorical | Yes/No |
| Female hormone use | Have you ever used female hormones such as estrogen and progesterone? Please include any forms of female hormones, such as pills, cream, patch, and injectables, but do not include birth control methods or use for infertility. | Categorical | Yes/No |

| **Supplementary Table 2.** Associations between CDAI Levels and the Risks of Endometriosis among all participants without female hormone use ^a^. | | | | | | |
| --- | --- | --- | --- | --- | --- | --- |
| **CDAI** | **Model A** | | **Model B** | | **Model C** | |
|  | **OR [95% CI]** | **p value** | **OR [95% CI]** | **p value** | **OR [95% CI]** | **p value** |
| As continuous (per SD) | 0.98[0.97,0.99] | 0.035 | 0.98[0.97,1.00] | 0.075 | 0.98[0.97,1.00] | 0.069 |
| Interquartile |  |  |  |  |  |  |
| Q 1 [-1.89,-1.79] | Reference | | Reference | | Reference | |
| Q 2 [-1.79,-0.69] | 0.94[0.84,1.05] | 0.265 | 0.92[0.83,1.03] | 0.162 | 0.93[0.82,1.04] | 0.223 |
| Q 3 [-0.69,1.42] | 0.99[0.89,1.10] | 0.900 | 0.97[0.87,1.08] | 0.606 | 0.97[0.86,1.09] | 0.663 |
| Q 4 [1.42,47.92] | 0.84[0.75,0.93] | 0.001 | 0.85[0.76,0.96] | 0.007 | 0.86[0.77,0.97] | 0.019 |
| p-trend | <0.001 | | 0.031 | | 0.050 | |
| Abbreviations: CDAI, composite dietary antioxidant index; CI, confidence interval; OR, odds ratio. ^a^ The associations between CDAI levels and the risks of and the Risks of Endometriosis are presented as ORs (95% CI). Model A adjusted for age. Model B adjusted for age, race, education level, marital status, the ratio of family income to poverty, number of live births and BMI. Model C further adjusted for alcohol use, smoking—cigarette use, hypertension, diabetes, congestive heart failure, coronary heart disease based on Model B. | | | | | | |

| **Supplementary Table 3.** Associations between CDAI Levels and the Risks of Endometriosis among all participants without antihypertensive medication use ^a^. | | | | | | |
| --- | --- | --- | --- | --- | --- | --- |
| **CDAI** | **Model A** | | **Model B** | | **Model C** | |
|  | **OR [95% CI]** | **p value** | **OR [95% CI]** | **p value** | **OR [95% CI]** | **p value** |
| As continuous (per SD) | 0.98[0.96,0.99] | 0.001 | 0.98[0.96,0.99] | 0.001 | 0.98[0.96,0.99] | 0.003 |
| Interquartile |  |  |  |  |  |  |
| Q 1 [-1.89,-1.79] | Reference | | Reference | | Reference | |
| Q 2 [-1.79,-0.69] | 0.99[0.90,1.09] | 0.862 | 0.978[0.888,1.08] | 0.642 | 1.00[0.905,1.11] | 0.989 |
| Q 3 [-0.69,1.42] | 0.99[0.90,1.09] | 0.936 | 0.976[0.886,1.07] | 0.618 | 1.01[0.913,1.12] | 0.835 |
| Q 4 [1.42,47.92] | 0.85[0.77,0.93] | <0.001 | 0.866[0.783,0.957] | 0.004 | 0.900[0.810,1.00] | 0.050 |
| p-trend | <0.001 | | 0.013 | | 0.087 | |
| Abbreviations: CDAI, composite dietary antioxidant index; CI, confidence interval; OR, odds ratio. ^a^ The associations between CDAI levels and the risks of and the Risks of Endometriosis are presented as ORs (95% CI). Model A adjusted for age. Model B adjusted for age, race, education level, marital status, the ratio of family income to poverty, number of live births and BMI. Model C further adjusted for alcohol use, smoking—cigarette use, diabetes, congestive heart failure, coronary heart disease based on Model B. | | | | | | |

| **Supplementary Table 4.** Associations between CDAI Levels and the Risks of Endometriosis among all participants without anticholesterol medication use ^a^. | | | | | | |
| --- | --- | --- | --- | --- | --- | --- |
| **CDAI** | **Model A** | | **Model B** | | **Model C** | |
|  | **OR [95% CI]** | **p value** | **OR [95% CI]** | **p value** | **OR [95% CI]** | **p value** |
| As continuous (per SD) | 0.98[0.96,0.99] | <0.001 | 0.98[0.96,0.99] | <0.001 | 0.98[0.96,0.99] | 0.002 |
| Interquartile |  |  |  |  |  |  |
| Q 1 [-1.89,-1.79] | Reference | | Reference | | Reference | |
| Q 2 [-1.79,-0.69] | 0.96[0.88,1.05] | 0.376 | 0.93[0.85,1.03] | 0.170 | 0.96[0.87,1.06] | 0.395 |
| Q 3 [-0.69,1.42] | 0.97[0.89,1.06] | 0.580 | 0.95[0.87,1.05] | 0.373 | 0.98[0.89,1.08] | 0.699 |
| Q 4 [1.42,47.92] | 0.83[0.76,0.91] | <0.001 | 0.84[0.76,0.92] | <0.001 | 0.87[0.79,0.96] | 0.008 |
| p-trend | <0.001 | | 0.003 | | 0.028 | |
| Abbreviations: CDAI, composite dietary antioxidant index; CI, confidence interval; OR, odds ratio. ^a^ The associations between CDAI levels and the risks of and the Risks of Endometriosis are presented as ORs (95% CI). Model A adjusted for age. Model B adjusted for age, race, education level, marital status, the ratio of family income to poverty, number of live births and BMI. Model C further adjusted for alcohol use, smoking—cigarette use, hypertension, diabetes, congestive heart failure, coronary heart disease based on Model B. | | | | | | |

| **Supplementary Table 5.** Associations between CDAI Levels and the Risks of Endometriosis among all participants without antidiabetic medication use ^a^. | | | | | | |
| --- | --- | --- | --- | --- | --- | --- |
| **CDAI** | **Model A** | | **Model B** | | **Model C** | |
|  | **OR [95% CI]** | **p value** | **OR [95% CI]** | **p value** | **OR [95% CI]** | **p value** |
| As continuous (per SD) | 0.98[0.97,0.99] | 0.001 | 0.98[0.97,0.99] | 0.001 | 0.981[0.969,0.992] | 0.001 |
| Interquartile |  |  |  |  |  |  |
| Q 1 [-1.89,-1.79] | Reference | | Reference | | Reference | |
| Q 2 [-1.79,-0.69] | 0.95[0.87,1.04] | 0.275 | 0.93[0.85,1.02] | 0.112 | 0.951[0.867,1.04] | 0.280 |
| Q 3 [-0.69,1.42] | 0.96[0.89,1.06] | 0.474 | 0.95[0.87,1.04] | 0.261 | 0.959[0.874,1.05] | 0.383 |
| Q 4 [1.42,47.92] | 0.85[0.77,0.92] | <0.001 | 0.85[0.77,0.93] | <0.001 | 0.866[0.787,0.952] | 0.003 |
| p-trend | <0.001 | | 0.003 | | 0.010 | |
| Abbreviations: CDAI, composite dietary antioxidant index; CI, confidence interval; OR, odds ratio. ^a^ The associations between CDAI levels and the risks of and the Risks of Endometriosis are presented as ORs (95% CI). Model A adjusted for age. Model B adjusted for age, race, education level, marital status, the ratio of family income to poverty, number of live births and BMI. Model C further adjusted for alcohol use, smoking—cigarette use, hypertension, congestive heart failure, coronary heart disease based on Model B. | | | | | | |

| **Supplementary Table 6.** Associations between CDAI Levels and the Risks of Endometriosis among all participants without missing value ^a^. | | | | | | |
| --- | --- | --- | --- | --- | --- | --- |
| **CDAI** | **Model A** | | **Model B** | | **Model C** | |
|  | **OR [95% CI]** | **p value** | **OR [95% CI]** | **p value** | **OR [95% CI]** | **p value** |
| As continuous (per SD) | 0.97[0.96,0.99] | <0.001 | 0.98[0.96,0.99] | 0.001 | 0.98[0.96,0.99] | <0.001 |
| Interquartile |  |  |  |  |  |  |
| Q 1 [-1.89,-1.79] | Reference | | Reference | | Reference | |
| Q 2 [-1.79,-0.69] | 0.95[0.87,1.04] | 0.276 | 0.94[0.86,1.03] | 0.201 | 0.95[0.86,1.04] | 0.266 |
| Q 3 [-0.69,1.42] | 0.94[0.86,1.03] | 0.224 | 0.94[0.86,1.03] | 0.198 | 0.95[0.86,1.04] | 0.305 |
| Q 4 [1.42,47.92] | 0.83[0.76,0.91] | <0.001 | 0.85[0.77,0.93] | <0.001 | 0.86[0.78,0.94] | <0.001 |
| p-trend | <0.001 | | 0.003 | | 0.005 | |
| Abbreviations: CDAI, composite dietary antioxidant index; CI, confidence interval; OR, odds ratio. ^a^ The associations between CDAI levels and the risks of and the Risks of Endometriosis are presented as ORs (95% CI). Model A adjusted for age. Model B adjusted for age, race, education level, marital status, the ratio of family income to poverty, number of live births and BMI. Model C further adjusted for alcohol use, smoking—cigarette use, hypertension, diabetes, congestive heart failure, coronary heart disease based on Model B. | | | | | | |
